# Photoelectrocatalytic Detection and Degradation Studies of a Hazardous Textile Dye Safranin T

**DOI:** 10.3390/nano13152218

**Published:** 2023-07-30

**Authors:** Muhammad Usman Sadiq, Afzal Shah, Jan Nisar, Iltaf Shah

**Affiliations:** 1 Department of Chemistry, Quaid-i-Azam University, Islamabad 45320, Pakistan; musmansadiq@chem.qau.edu.pk; 2 National Center of Excellence in Physical Chemistry, University of Peshawar, Peshawar 25120, Pakistan; jan_nisar@uop.edu.pk; 3 Department of Chemistry, College of Science, United Arab Emirates University, Al Ain P.O. Box 15551, United Arab Emirates

**Keywords:** electrochemical sensor, safranin T, hazardous dye, nanomolar detection, photocatalytic degradation, nanomaterials

## Abstract

Herein, we report an electrochemical scaffold consisting of functionalized multiwalled carbon nanotubes (COOH-*f*MWCNTs) and iron-doped zinc oxide nanoparticles (Fe-ZnO) for the detection of a hazardous textile dye safranin T (ST) and monitoring of its photocatalytic degradation. Prior to the detection and degradation analysis, Fe-ZnO NPs were synthesized by the sol-gel method and characterized by a number of structural and morphological techniques. The carboxyl moiety of COOH-*f*MWCNTs possessing a strong affinity for the amino functionality of ST led to significant enhancement of the current response at the designed electrochemical platform, whereas the electrocatalytic role, surface area enhancement, and the provision of binding sites of Fe-ZnO led to a further increase in the peak current intensity of ST. Electrochemical impedance spectroscopy showed that the sensing scaffold made of the glassy carbon electrode modified with COOH-*f*MWCNTs and Fe-ZnO efficiently transfers charge between the transducer and the redox probe. Under optimized conditions, the developed sensor showed a 2.3 nM limit of detection for ST. Moreover, recovery experiments and anti-interference tests qualified the sensing platform for practical applications. The dye was photocatalytically degraded using Fe-ZnO NPs up to 99% in 60 min with a rate constant of 0.068 min^−1^. The designed sensor was used to probe the degradation kinetics of the target dye, and the results were found consistent with the findings obtained from electronic absorption method. To the best of our knowledge, the present work is the first approach for the efficient detection and almost absolute degradation of ST.

## 1. Introduction

Water is crucial for the survival of biota on earth. Industrial effluents released into water bodies without any proper treatment change the taste, color, and pH of water [1,2,3]. Industrial waste possessing radioactive materials, hazardous chemicals, toxic gases, heavy metals, and organic contaminants renders water and air unhealthy to drink and breathe [4,5,6]. Among a variety of water contaminants, dyes are found in abundance owing to their use in paper, tanning, textile, cosmetics, plastic, and pharmaceuticals [7,8,9]. The untreated discharge of dyes loaded effluents reduce photosynthesis in aqueous ecosystems leading to the extinction of aquatic biota [10,11]. A number of dyes have been reported to cause jaundice, tumors, skin infections, and allergic reactions [12,13,14,15]. Some dyes are carcinogenic and mutagenic [16,17].

Safranin T (ST) is a basic dye that belongs to the class of azine dyes. It is widely used in paper, silk, wool, leather, mordanted cotton, tannin, and food industries [18]. It also finds use as a photosensitizer, biological stain, and fluorescent probe [19,20]. ST is associated with several health issues including eye irritation, skin infections, and respiratory tract irritation. When swallowed, it causes severe digestive infections such as nausea, vomiting, and diarrhea [21]. China has prohibited the use of ST as a food ingredient [22]. The European Community has imposed limits of 0.5–1.0 mg kg^−1^ on all carcinogenic dyes [23]. Therefore, it is imperative to develop an effective and environmentally benign method for the detection and removal of toxic dyes. The literature reveals that various detection approaches such as super high-performance liquid chromatography (SHPLC) [24], fluorescence spectroscopy [25], the spectrophotometric method [26,27,28,29], ultrasound-assisted dispersive solid-phase micro extraction [30], and surface-enhanced Raman spectroscopy [31] are used for the determination of ST. However, low sensitivity, complicated and expensive apparatus, and the use of poisonous materials limit practical applications of the methods. Therefore, researchers are searching for a simple, fast, eco-friendly, and selective method for ST detection. In this regard, electrochemical methods have emerged as an alternative ultrasensitive detection method due to their easy handling, promising selectivity, sensitivity, low cost, environmental friendliness, and fast responsiveness [32,33]. Owing to its widespread use as a colorant in the textile and food sectors, ST is regarded as a model compound mimicking the dyes discharged into the wastewater of these sectors. The presence of electron-rich moiety and conjugation in ST make it a suitable analyte for detection via electrochemical techniques. Therefore, the current work presents an electrochemical sensor made of a glassy carbon electrode modified with functionalized multiwalled carbon nanotubes (COOH-*f*MWCNTs) and iron-doped zinc oxide nanoparticles (Fe-ZnO) for the detection and degradation monitoring of ST. The reasons for the use of COOH-*f*MWCNTs and Fe-ZnO in the designed sensing platform are presented in the subsequent paragraphs.

Carbon nanotubes (CNTs) are appealing materials for a variety of applications; however, their limited dispersibility and solubility in organic and aqueous media hamper their applications [34,35]. To overcome these issues, CNTs are functionalized by various methods [36]. These functionalization strategies not only improve dispersibility but also give unique properties to the CNTs’ structure [37]. The derivatization of CNTs with carboxyl moieties boosts the dispersity of these materials by disrupting the hydrogen bonding on their surface. The functionalization of CNTs with carboxyl moieties improves the lyophilicity as well as the dispersibility of CNTs in various solvents, making them promising for sensing applications. The lyophilic nature of COOH-*f*MWCNTs is susceptible to analyte adsorption, which makes them efficient sensors in comparison to simple MWCNTs. Electrochemical sensors based on COOH-*f*MWCNTs have been reported for a variety of analytes [38,39]. The coupling of CNTs with nanoparticles has been reported to further enhance the sensing characteristics of the modified electrode surface, which can be attributed to the provision of greater active surface area for analyte adsorption at their surface [40]. 

Zinc oxide is an important semiconducting material that has a 3.37 eV band gap energy and an almost 60 meV excitons binding energy in ambient conditions [1]. It finds usage in transparent conductors, photocatalysis, chemical sensors, optical, and piezoelectric devices. The applications of ZnO have a strong dependence on its size, aspect ratio, and morphology [41,42]. Numerous investigations have been conducted to modify the attributes of ZnO NPs to make them appropriate for various applications. Specific characteristics, including optical, magnetic, electrical, and structural properties of ZnO, can be modulated by selectively doping it with various elements, especially transition metal ions. ZnO is regarded as an excellent host for doping with various elements. Doping can significantly raise the charge carrier count in semiconductors resulting in improved conductivity compared to pristine material [43]. Along with the improvement of ferroelectric and ferromagnetic features, doping of ZnO with metal ions improve its sensing capabilities due to the production of additional electron-hole pairs and enhanced surface area [1]. Fe-ZnO nanostructures have been widely utilized for catalysis, ferromagnetism, UV photodetectors, and sensing applications [44,45]. The use of Fe^3+^ as a dopant is favorable due to the similarity in electronegativity and ionic radii of Fe^3+^ and Zn^2+^ [46]. This can improve the electronic/ionic conduction of doped material in comparison to undoped one. Therefore, in the present study, COOH-*f*MWCNTs and Fe-ZnO NPs have been used as a recognition layer for the alteration of the electrode surface owing to their improved electrochemical properties. Moreover, in addition to detection objectives, Fe-ZnO NPs were also used as photocatalysts for the degradation of ST.

In developing countries, rapid urbanization and fast-growing industries have adverse impacts on ecology and human health. Oil, gas, textile, and other industries release billions of tons of chemicals every year. Textile dyes comprise a major portion of pollution [47,48]. The buildup of dyes in water reservoirs results in eutrophication, depletes reoxygenation ability, and badly impacts aquaculture by obstructing sunlight infiltration [49]. Several methods such as photocatalysis, ozonation, electrocatalysis, membrane coagulation, microbial, and fungal adsorption methods have been utilized to eradicate common organic and inorganic contaminants [50,51]. Among these approaches, heterogeneous photocatalysis has attracted much interest in recent years. Kemary et al. reported that zinc sulphide NPs photodegrade 51% ST in 40 min [52]. Janaki et al. evaluated the photocatalytic activity of different semiconducting materials for the degradation of ST. They achieved 99% photodegradation using TiO_2_ NPs, 80.3% using ZnO, and 57.1% utilizing bismuth oxide nanoparticles while all other tested materials showed less than 20% degradation [53]. Hammad et al. employed biosynthesized copper oxide NPs for the photodegradation of ST and achieved 63% degradation in two hours [54]. ZnO NPs have been recognized as excellent photocatalysts for the removal of dyes, and their photocatalytic efficacy can be further enhanced by doping. The introduction of iron as a dopant hinders the recombination of electron–hole pairs in visible light, thus the photocatalytic activity of Fe-ZnO improves in comparison to pristine ZnO under sunlight illumination. Therefore, we opted for Fe-ZnO as a catalyst for the photocatalytic degradation of ST, which degraded it up to 99% in 60 min.

In this study, we report an efficient and environmentally friendly method for the sensitive detection of ST. An electrochemical sensing layer comprising COOH-*f*MWCNTs and Fe-ZnO was coated over the transducer for the detection of ST using square-wave voltammetry (SWV). Fe-ZnO NPs were utilized for the photocatalytic degradation of ST. The prepared sensor was also employed for the electrochemical monitoring of photocatalytic degradation of ST along with spectrophotometric studies. Our devised novel method involves the immobilization/deposition of the analyte directly on the surface of the modified electrode, rather than taking it in the conventional electrochemical cell. This method is environmentally friendly since it requires a very small amount of the dye during experimental work, while in the case of the conventional solution approach, a large amount of analyte is used, which in most cases, is discarded without any treatment. It also allows closer accessibility of the analyte to the sensing layer, which results in the generation of an intense voltammetric signal by minimizing the orientation, as well as a diffusion barrier of the analyte dissolved in solution taken in the conventional electrochemical cell. Hence, the adopted method of analyte immobilization at the electrode surface not only allows sensitive detection of ST but also allows the monitoring of degradation studies. The present study is the first report on achieving the dual objectives of detection and degradation monitoring of ST using the designed electrochemical sensing platform.

## 2. Experimental

### 2.1. Materials

Analytical-grade COOH-*f*MWCNTs and ST were procured from Sigma-Aldrich. Zinc nitrate hexahydrate, iron nitrate hexahydrate, ethanol, and sodium hydroxide of analytical quality were used for the synthesis of ZnO and Fe-ZnO NPs. All other chemicals/reagents utilized during this work as supporting electrolytes, for pH maintenance, and as interfering agents are listed in Table S1 along with their purity and supplier.

### 2.2. Instrumentation

The surface morphology and elemental composition of synthesized materials were evaluated using scanning electron microscopy (SEM) and energy dispersive X-ray (EDX) analysis utilizing the JOEL-JSM-IT100 instrument operating at 20 kV. The optical properties of ZnO and Fe-ZnO were explored using a Shimadzu (UV-1700) spectrophotometer. X-ray diffraction (XRD) analysis was performed in an X-ray diffractometer (Philips, X’Pert Pro Super, CuKα = 1.540589 Å). Metrohm Autolab (Galvanostat/Potentiostat) (Utrecht, The Netherlands) running with NOVA 1.11.0 software was employed for voltammetric measurements, i.e., cyclic voltammetry (CV), square wave voltammetry (SWV), and electrochemical impedance spectroscopy (EIS).

### 2.3. Synthesis Route of ZnO and Fe-ZnO NPs

**Scheme 1:**
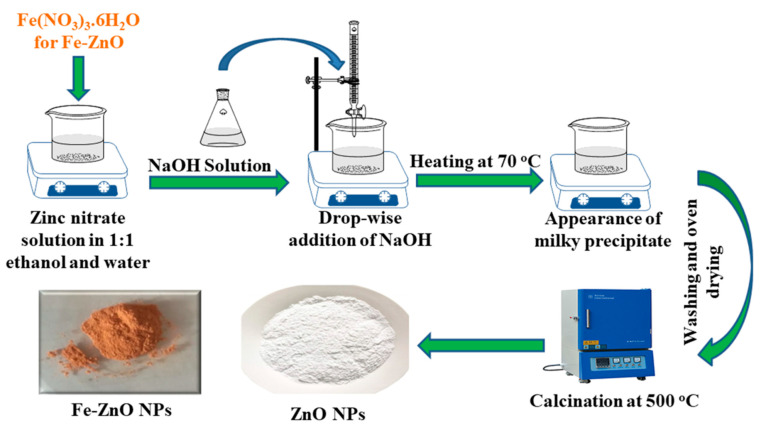
Sol–gel fabrication of ZnO and Fe-ZnO nanoparticles.

To fabricate ZnO and Fe-ZnO NPs, the sol–gel method was adopted (Scheme 1). In this procedure, 1 g of zinc nitrate hexahydrate was dissolved in 50 mL of 1:1 water and ethanol mixture, which was stirred for approximately 10 min to obtain a clear solution. A 4% weight/volume solution of NaOH was prepared by dissolving 2 g of NaOH in distilled water with a total volume of 50 mL. The as-prepared 50 mL NaOH solution was added dropwise using a burette into a zinc nitrate hexahydrate solution while being vigorously stirred. After the complete addition of the NaOH solution, the mixture was heated at 70 °C until the appearance of a milky precipitate. The precipitates were then washed with ethanol followed by washing thrice using distilled water until the neutral pH was attained. The obtained white precipitates were then oven-dried at 80 °C followed by calcination at 500 °C for 3 h. The same strategy was employed for the synthesis of Fe-ZnO nanoparticles except that along with zinc nitrate hexahydrate, a stoichiometric amount of iron nitrate hexahydrate was also added.

### 2.4. Fabrication of Modified Electrode

The surface cleanliness of GCE is very crucial in electrochemical analysis. Before the electrochemical analysis, GCE was cleaned until the appearance of a shiny smooth surface. The physiochemical cleaning of GCE was performed utilizing a nylon rubbing mat. The GCE was rubbed on a rubbing mat containing aqueous alumina slurry with a 1 µm particle size. The surface of GCE was gently rubbed on the rubbing mat in the pattern of a figure eight, producing a smooth appearance of GCE. The physiochemical-cleaned GCE was sonicated in an ethanol, acetone, and water mixture for 10 min followed by drying in ambient conditions. The sonication of GCE in a solvent mixture might help to remove the contaminants attached to the surface during polishing. Following that, GCE was electrochemically cleaned by recording cyclic voltammograms in a 0.1–0.5 V potential window until reproducible results were achieved.

For the fabrication of modified GCE, 1 mg/1 mL dispersions of COOH-*f*MWCNTs and nanoparticles were prepared in dimethylformamide via 3-h ultrasonication. The pre-cleaned GCE was modified by depositing 5 µL of Fe-ZnO and 5 µL of COOH-*f*MWCNTs using a layer-by-layer approach, followed by air drying. This modified GCE was deployed to detect ST. For electrochemical measurements of ST, a 0.1 mM solution was made using distilled water. It was further diluted using distilled water to obtain the desired concentration of an analyte. After the fabrication of the modified GCE, a 10 µL droplet of the 30 µM analyte solution was drop cast on the modified electrode, followed by air drying of the analyte. After ensuring the complete drying of GCE, an electrochemical cell was set using the phosphate buffer as an electrolyte to record the electrochemical behavior of ST at modified GCE. The cell assembly used for electrochemical measurements was primarily made up of a three-electrode system and an electrochemical cell. The electrochemical cell was made up of double-walled glass mounted with a Teflon cell top. The cell top is typically a self-mounting easily detachable teflon cap containing five holes. Three holes are utilized for the insertion of three electrodes (reference, working, and auxiliary electrodes), whereas the remaining two holes are utilized for purging purposes (outlet and inlet for gas). The volume capacity of the electrochemical cell was 20 mL. The capability of the developed sensor to sense ST was assessed by executing SWV and comparing their responses with that obtained at bare GCE. A detailed scheme of the voltammetric measurements is presented in Scheme S1. CV and EIS were also executed for the developed sensor using a redox probe potassium ferricyanide to study the charge transfer ability of the modifier. 

### 2.5. Dye Degradation Procedure

The photocatalytic degradation of ST was monitored using electrochemical (SWV) and UV-visible spectroscopy techniques. For photodegradation studies, the ST solution was placed under direct sunlight. A 1 mM stock solution of ST was prepared using distilled water, which was further diluted to 80 µM. For photodegradation, pH optimization was carried out using 0.1 M HCl and 0.1 M NaOH solution. Prior to the start of the photodegradation experiment, a 2 mg amount of catalyst was added to the 40 mL of ST solution and kept under stirring in the dark for 20 min to attain adsorption-desorption equilibrium. ST solution containing catalyst was then placed in the sunlight. Two milliliters of the ST sample were collected using a micropipette after a specific time interval, and degradation was monitored using UV-visible spectroscopy. For voltammetric monitoring of degradation, a 50 µL sample was collected in a vial and placed in the dark to stop the photoreaction, from which a 10 µL drop was then immobilized at the modified electrode for SWV measurements. 

## 3. Results and Discussion

### 3.1. Characterization of Pure ZnO and Fe-ZnO NPs

**Figure 1:**
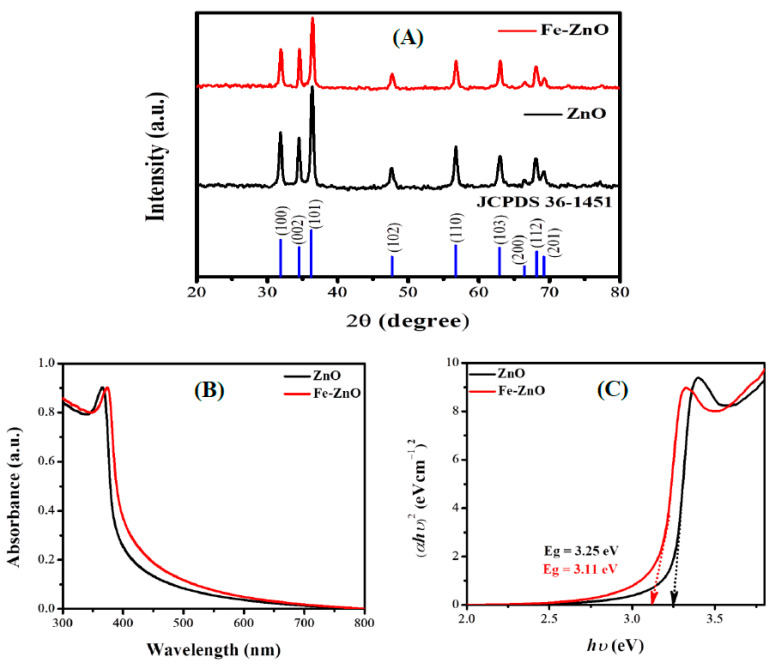
(**A**) XRD spectra of pure and Fe-ZnO NPs; (**B**) UV-visible absorption spectra of pristine ZnO and Fe-ZnO NPs; (**C**) Tauc plots of ZnO and Fe-ZnO NPs.

The X-ray diffractograms of pristine ZnO and Fe-ZnO NPs are presented in Figure 1A. All observed XRD peaks are in good agreement with the reported literature (JCPDS 36-1451), corresponding to a pure hexagonal wurtzite structure [40]. The XRD pattern of Fe-ZnO NPs showed no additional peaks, which indicates that Fe ions are well integrated into the Zn crystal lattice without producing any disturbance in the crystal structure of ZnO NPs, and no evidence of impurities of the secondary phase (Fe_2_O_3_ and pure Fe) is observed. The diffraction peak intensity of Fe-ZnO is reduced compared to pure ZnO, which indicates a reduction in crystallinity due to lattice strain and disorder resulting from the replacement of Zn by Fe ions. Similarly, the diffraction peaks are observed to shift to a higher angle, which represents the lattice expansion and reduction in d-spacing [55]. 

Fe ions have two oxidation states (Fe^3+^ and Fe^2+^). Because of their different charge and ionic radii (Fe^3+^ 0.078 nm and Fe^2+^ 0.068 nm), they both exert distinctive impacts on structural characteristics. In the present case, the shift of diffraction peaks towards the right side of the spectrum resulting from compressional strain in the crystal structure of ZnO shows that Fe ions primarily exist in Fe^3+^ form. This may be related to the reduced ionic radii of Fe^3+^ (0.068 nm) compared to Zn^2+^ (0.072 nm). The average crystallite size of pristine ZnO and Fe-ZnO NPs was determined by Debye–Scherrer’s relation.

(1) $$D=\frac{K\lambda }{\beta cos\theta }$$

where *D* represents the average crystallite size of particles, *K* (0.94) denotes the Scherrer constant, *λ* is the X-ray wavelength (CuKα = 0.15406 nm), *β* represents the peak full-width half maximum (FWHM), and *θ* is the peak position (Braggs angle). The average crystallite size was found to be 16.18 nm and 18.92 nm for ZnO and Fe-ZnO NPs, respectively.

UV-visible absorption spectra of undoped ZnO and Fe-ZnO are depicted in Figure 1B. The absorption spectrum of ZnO shows its characteristic absorption maxima at 365 nm, whereas a bathochromic effect is observed for Fe-ZnO with an absorption peak at 373 nm. The observed red shift in wavelength can be attributed to the additional energy level that arises from the Fe^3+^ doping in the ZnO crystal lattice resulting in the depletion of the band gap. The band gap value was estimated using the following equation.

(2) (αhυ)1n=A(hυ−Eg)

where “*h*” represents the Planck’s constant, “*A*” is the proportionality constant, “*α*” is the absorption coefficient, “*υ*” is the frequency of photons, and “*E_g_*” signifies the band gap energy of ZnO and Fe-ZnO NPs. The direct transition band gap has a value of 1/2 for “*n*”, while it has a value of 2 for the indirect band gap. The determined band gap for ZnO and Fe-ZnO is depicted in Figure 1C, which shows a reduction in band gap value after doping. The band gap of ZnO (3.25 eV) was tuned by the inclusion of Fe^3+^ ions into the ZnO crystal structure to 3.11 eV. The band gap of ZnO NPs was reduced after doping with iron ions. The decrease in the band gap may occur due to an increased concentration of charge carriers, which may result in the inclusion of extra energy levels above or below the conduction band. The doping can be interstitial or substitutional. For interstitial doping, the size of the dopant must be very small in comparison to the host size so that it can easily diffuse into the interstitial sites. Whereas in the case of substitutional doping, the size of the dopant must be comparable to the host size. In the present case, as revealed by the XRD data, the Fe ions are mostly present as Fe^3+^, the size of which is identical to the Zn. Therefore, we can speculate that substitutional doping occurs in the present case.

**Figure 2:**
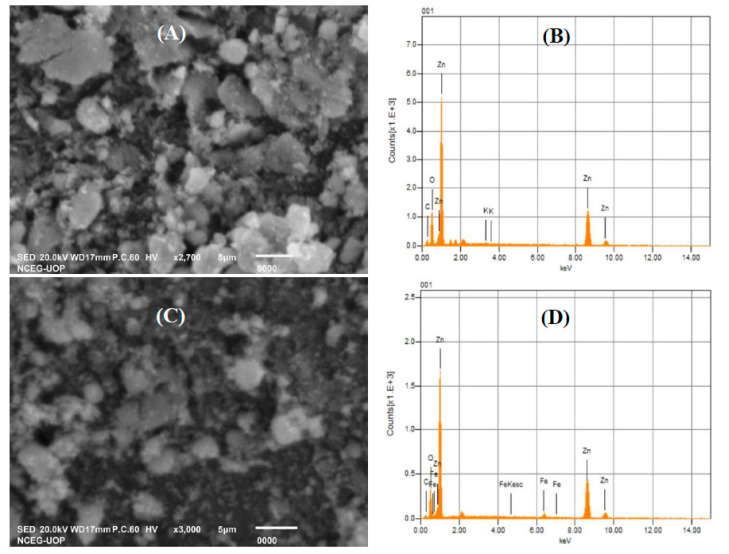
(**A**) SEM images of ZnO and (**C**) Fe-ZnO NPs; EDX plots of (**B**) ZnO and (**D**) Fe-ZnO NPs.

The surface properties of pristine and doped ZnO samples were determined using SEM analysis (Figure 2A,C). SEM images of ZnO NPs (Figure 2A) display the agglomeration of NPs having irregular morphologies. The NPs aggregated to form sheet-like aggregates, in addition to spherical shapes, which are in minor numbers. However, the Fe-ZnO sample shows less aggregation of NPs having quasi-spherical morphology (Figure 2C). In addition to reduced agglomeration, a decrease in particle size can be visualized; however, exact particle size determination is a strenuous task owing to particle aggregation.

The elemental formulation and purity of pure ZnO (Figure 2B) and Fe-ZnO NPs (Figure 2D) were evaluated utilizing EDX analysis. EDX results confirmed the presence of Zn, Fe, and O as the major constituents of samples. A trace amount of C was also detected, which arise due to the carbon ribbon used during EDX analysis. The detailed elemental makeup of ZnO and Fe-ZnO NPs is presented in Table S2. These findings also indicate the absence of any contaminants.

### 3.2. Electrochemical Characterizations

EIS is a useful approach to investigate the interfacial characteristics of modified and bare GCE. The impedance spectrum consists of two parts, a semicircular part at high frequencies and a linear part at low frequencies. The linear section represents the Warburg impedance (Z_w_), while the semicircular part shows the charge transfer resistance (R_ct_). Z_w_ and R_ct_ depict the mass transport and electron transfer at the electrode–solution interface [9].

**Figure 3:**
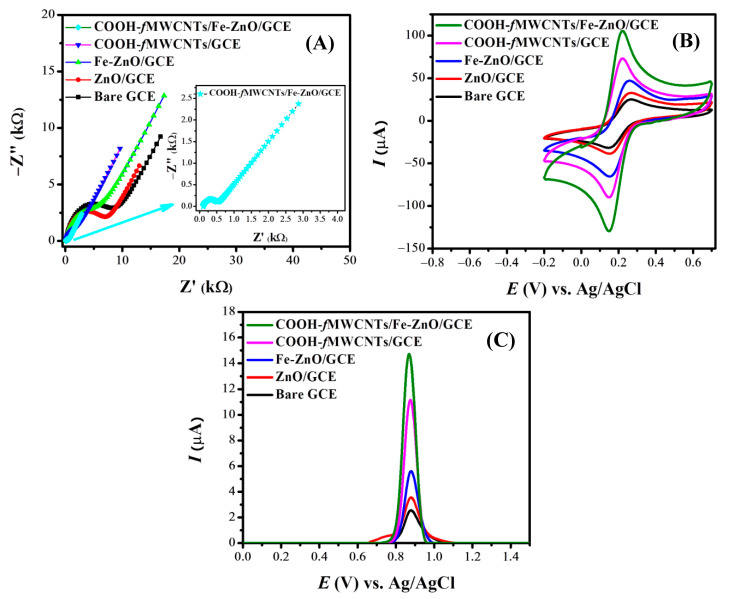
(**A**) Nyquist curves of bare and modified GCEs containing 5 mM K_3_[Fe(CN)_6_] and 0.1 M KCl solution; (**B**) cyclic voltammograms recorded with bare and modified GCEs for 5 mM M K_3_[Fe(CN)_6_] containing 0.1 M KCl as an inert electrolyte; (**C**) square wave voltammograms of 30 μM ST at bare GCE and modified GCEs in 0.1 M PBS (pH = 7).

EIS measurements were conducted using 0.1 M KCl and 5 mM K_3_[Fe(CN)_6_] solution by varying the frequency from 1 MHz to 0.1 Hz with 10 mV amplitude. Figure 3A depicts the Nyquist plots obtained using the fitted data collected from EIS responses of the modified and bare electrodes. The bare GCE exhibited a larger semicircular part with an R_ct_ value of 8.03 kΩ, indicating a higher interfacial resistance between bare GCE and electrolyte. The semicircular portion after modification shows a sequential decrease in R_ct_ values with the minimum value of 395.3 Ω for COOH-*f*MWCNTs/Fe-ZnO/GCE. The R_ct_ of COOH-*f*MWCNTs/Fe-ZnO/GCE as shown in the inset of Figure 3A is significantly lower than the unmodified electrode, demonstrating a rapid electron transfer process, owing to enhanced electrochemical performance. For parameter assessment, EIS data were fitted using Randles’ equivalent circuit. Figure S1 depicts the equivalent circuit model (RCRW) used to fit the experimental data, which includes a Warburg impedance, resistor, and constant phase element. The fitted impedance data for bare and modified GCEs are listed in Table S3.

To probe the voltammetric behavior of bare and modified GCEs, cyclic voltammetry was executed by using 5 mM K_3_[Fe(CN)_6_] as a redox probe and 0.1 M KCl as an inert electrolyte at a 0.1 Vs^−1^ scan rate in the potential window from −0.2 V to 0.7 V. Figure 3B shows the electrochemical responses of bare GCE and modified GCEs, which shows an increase in current responses in comparison to bare GCE. Cyclic voltammograms reveal that the anodic peak on COOH-*f*MWCNTs/Fe-ZnO/GCE shifted towards lower potential of 217 mV in comparison to 268 mV, 263 mV, 258 mV, and 222 mV of bare GCE, ZnO/GCE, Fe-ZnO/GCE, and COOH-*f*MWCNTs/GCE with significant enhancement of peak current. A decrease in peak separation ∆*E_p_* = 65 mV was observed for COOH-*f*MWCNTs/Fe-ZnO/GCE compared to others. The shift of the peak potential towards a lower value, improvement in reversibility, and remarkable increase in current show that the modified electrode eases the redox processes.

The surface area of the electrode plays a key role in the electroanalytical behavior of the sensor. An increase in the electrode surface area leads to an increased number of binding sites available for the analyte, resulting in the robustness of the analyte signal. The active surface area of modified and bare electrodes was ascertained using the Randles–Sevcik equation shown in Equation (3), by using the peak current of cyclic voltammograms.

*I_pa_* = 2.6 × 10^5^ *n*^3/2^*D*^1/2^*ν*^1/2^*A* (3)

where *I_pa_* denotes the oxidation peak current measured in Ampere, *A* is the electroactive surface area of the electrode in cm^2^, *D* is the diffusion coefficient in cm^2^s^−1^, *υ* is the scan rate in Vs^−1^, *n* is the number of electrons, and *C* is the concentration of the probe solution in mol cm^−3^. The calculated surface area and peak separations of modified and bare electrodes are shown in Table S4. COOH-*f*MWCNTs/Fe-ZnO/GCE exhibits a fourth-fold increase in surface area in comparison to bare GCE. The increased surface area offers more binding sites for the analyte molecules, which facilitates faster electron transfer. Furthermore, minimum peak separation and the highest peak current reflect the enhanced electrocatalytic performance of COOH-*f*MWCNTs/Fe-ZnO/GCE compared to bare GCE.

### 3.3. Voltammetric Behaviour of Safranin T

To evaluate the performance of the designed modifiers towards the oxidation of ST, SWV measurements were conducted using a phosphate buffer solution (PBS) of pH 7. Voltammograms were acquired using a 30 µM ST solution in a potential window of 0 V to 1.5 V keeping an accumulation potential of 0.1 V and an accumulation time of 5 s. Figure 3C depicts the SWV responses of modified and bare electrodes toward the oxidation of ST. COOH-*f*MWCNTs/Fe-ZnO/GCE demonstrates maximum anodic current response for ST in comparison to COOH-*f*MWCNTs/GCE, Fe-ZnO/GCE, ZnO/GCE, and bare GCE. As COOH-*f*MWCNTs/Fe-ZnO/GCE demonstrates the minimum R_ct_ value and maximum active surface area compared to bare and other modified electrodes, the current response of ST is notably multi-fold higher for the developed sensor. Due to the enhanced electroactive surface area, adsorptive sites on the modified surface are easily accessible, indicating that the carboxylic group is easily accessible to the amino group of SST, which leads to an acid–base-type interaction. Akin to this, when Fe-ZnO particles are used in conjunction with COOH-*f*MWCNTs, Fe-ZnO particles facilitate the transport of electrons between COOH-*f*MWCNTs/Fe-ZnO/GCE and analyte molecules due to the availability of more adsorptive sites, which permits the attachment of an increased number of analytes to the GCE surface. These findings are in good agreement with SEM results and band gap energy values. Since the Fe-ZnO NPs are less agglomerated compared to ZnO, more surface area is available for the attachment of analyte molecules on the modified electrode surface and the reduction in band gap further facilitates the facile electron transfer. In comparison to other investigated materials, COOH-*f*MWCNTs/Fe-ZnO exhibits the maximum current for the given dye, owing to the acid–base-type interaction between the analyte and the modifier, and electronic modification due to defects created by Fe-ZnO, which allows faster electron transfer to the electrode surface. Therefore, COOH-*f*MWCNTs/Fe-ZnO was chosen as the optimum catalyst for the sensing of ST.

### 3.4. Effect of Scan Rates

CV is a promising analytical tool to deduce the nature of an electrochemical reaction, whether it is diffusive or adsorptive. To ascertain the type of the reaction, cyclic voltammograms were recorded at various scan rates, and then the plot of the log of peak current versus the log of scan rate was used to determine the nature of the reaction. A review of the literature shows that if the slope of the plot between log *I_p_* versus log υ is 0.5 or greater, the process should be governed by diffusion, and if it is closer to 1, it should be controlled by adsorption [13]. The effect of scan rates on the electrocatalytic behavior of COOH-*f*MWCNTs/Fe-ZnO/GCE towards the oxidation of ST was studied in 0.1 M PBS solution. Figure S2A shows the CV responses of ST oxidation, recorded at various scan rates spanning from 25 mVs^−1^ to 150 mVs^−1^ With an increase in scan rate, the peak current of ST oxidation steadily rises, while the peak potential at higher scan rates is slightly shifted to a higher potential. This positive shift in peak potential is attributed to the irreversible nature of ST oxidation and can also be related to the thickness of the diffusion layer. At higher scan rates, a substantially thinner diffusion layer is produced in contrast to low scan rates, which results in a thicker layer that grows significantly farther away from the surface of the electrode. As a result, the surface of the electrode experiences a noticeably lower changing potential at a reduced scan rate, resulting in the observed potential shift [39]. Similarly, the reverse scan shows no cathodic peak for ST reduction, indicating the irreversible nature of the reaction. The plot between log *I_p_* vs. log υ shows a slope value of 0.82 (Figure S2D) indicating the involvement of both adsorption and diffusion processes in the reaction. However, a higher correlation factor of the plot of scan rate versus peak current (Figure S2B), compared to the plot between peak current and square root of scan rate (Figure S2C), shows that the adsorption process is favorable at the surface of the modified electrode.

### 3.5. Optimization of Experimental Parameters

An experimental setup employs several distinct experimental parameters, and changing these variables has a strong influence on the experiment’s outcomes. Electrochemical experiments also have a variety of factors that control the peak current (*I_p_*), peak shape, and peak potential (*E_p_*) of analytes. The most important experimental parameters in voltammetric experiments are the selection of a suitable electrolyte, the concentration or pH of the electrolyte, accumulation potential, and accumulation time. After confirming the sensing ability of the devised sensor for the targeted analyte, the above-mentioned parameters were optimized to attain the best possible value of peak current.

The nature of supporting electrolytes affects the voltammetric response of the analyte. The Ohmic drop, migration current, and ionic strength of the solution depend strongly on the electrolyte [38]. Therefore, the effects of supporting electrolytes on the response of COOH-*f*MWCNTs/Fe-ZnO/GCE towards the oxidation of ST were examined using a variety of electrolytes including basic (NaCl, NaOH), acidic (H_2_SO_4_, HCl), buffer (Britton–Robinson buffer of pH 7, citrate buffer saline of pH 6, phosphate buffer of pH 7), and neutral salt (KCl, KNO_3_) solutions. Figure S3 illustrates the substantial impact of media on the response of ST. The COOH-*f*MWCNTs/Fe-ZnO/GCE sensor showed a well-defined and intense peak in a PBS (pH = 7) buffer solution in comparison to other tested supporting electrolytes. Therefore, PBS was selected as the best working medium for the rest of the electroanalysis of ST.

**Figure 4:**
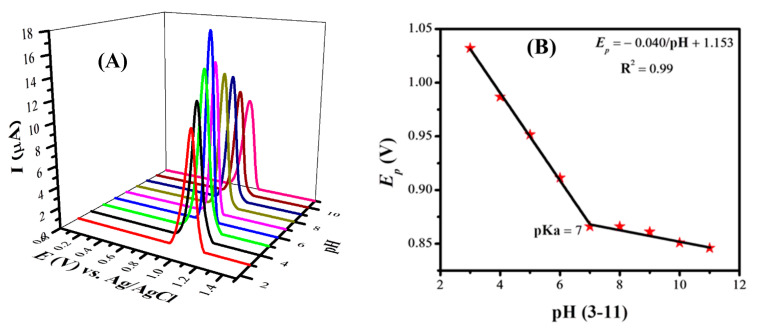
(**A**) SWV curves of 30 µM ST in PBS solution at different pH values (3–11) recorded using COOH-*f*MWCNTs/Fe-ZnO/GCE as a sensor; (**B**) plot of *E_p_* (V) of ST oxidation as a function of pH (3–11) of phosphate buffer.

The pH of the solution is important in assessing the analyte detection performance of the sensor. Proton transfers that might take place throughout the electrode process may affect the pH and rate of the electro-oxidation processes. The fluctuation in the magnitude of the analyte signal results from the variation in dissociation constants of the base and acid. Additionally, the pH of the working medium influences the availability of proton, ionization of functional groups of the modifier, and charge transduction at the surface of the electrode. Therefore, any variation in the pH of electrolytes leads to an obvious impact on the peak shape, current, and potential. To evaluate the impact of pH on the anodic peak of ST (30 µM), SWVs were recorded by varying the pH of PBS in the range of 3–11. The SWVs of ST oxidation at different pH is depicted in Figure 4A. A gradual rise in current with an increase in the pH of the PBS buffer was observed up to a pH value of 6, and then a decline in current value occurred. The optimum value of current for ST oxidation was achieved at pH 6 (Figure S4). Figure 4B shows the strong dependence of peak potential on pH. Peak potential displays reciprocity with pH up to a pH value of 7, and beyond this, a slight variation in potential (nearly similar) was observed for pH 8.0, 9.0, 10.0, and 11.0. The value of peak potential decreased as the pH rose, inferring the engagement of protons in the redox process. The slope value (40 mV/pH) of the plot of *Ep* versus pH shows deviation from the Nernstian value (59 mV/pH) indicating the engagement of an unequal number of protons and electrons in the oxidation of ST. The flex point of the plot delineates the pKa (acid–base dissociation constant) of ST, which is in good agreement with the reported values. 

The influence of accumulation potential (E_d_) on the voltammetric oxidation response of ST at the developed sensor was probed using a PBS (pH 6) solution. Dye molecules are mostly bulkier, which causes steric hindrance to their proper orientation and thus affects their electrochemical behavior. Proper orientation of the analyte molecules at the electrode surface is a decisive factor to obtain an optimized outcome because the maximum number of analyte molecules are presumed to direct their electroactive groups to the surface of the sensing scaffold for the redox processes. The influence of E_d_ variation from −0.4 V to 0.4 V is presented in Figure S5. The increment in the anodic peak intensity of ST was found with the rise of accumulation potential until −0.2 V. Beyond this potential value, a reduction in *I_p_* was observed owing to the saturation of active sites. Therefore, −0.2 V E_d_ was selected for subsequent electrochemical studies of ST due to its highest peak current response. The maximum current observed at a negative potential (−0.2 V) can be related to the cationic nature of ST, which may interact strongly with the sensing scaffold at this potential. The deposition time (D_t_) is also an important parameter that significantly influences preconcentrating the analyte on the surface of the sensor. The accumulation of the analyte mostly increases with increasing D_t_, as more dye molecules become able to properly orientate themselves at the modified surface of the electrode, which results in the generation of an intense current signal. After the saturation level of the electrode surface, a decrease in current occurs due to the increased thickness of the layer of analyte molecules, which may hinder further mass transport and electron transfer between the analyte and transducer. The impact of deposition time on peak intensity was evaluated by varying the time duration from 5 to 25 s at −0.2 V accumulation potential. The maximum peak intensity of the dye was observed at a D_t_ of 10 s (Figure S6). When the D_t_ surpassed 10 s, the amount of dye that could be oxidized on the modified electrode approached a limiting value, as indicated by a decline in current value. It can be ascribed to the saturation of all accessible active sites at the sensor surface with electro-oxidized dye. Consequently, when the accumulation duration increases, the thickness of dye layers at COOH-*f*MWCNTs/Fe-ZnO/GCE also increases, which obstructs further mass transport of dye molecules. Thus, 10 s was opted as an optimized value of time for further studies of ST oxidation.

### 3.6. Analytical Application of Developed Sensing Scaffold

#### 3.6.1. Evaluation of Quantification and Detection Limits of ST

**Figure 5:**
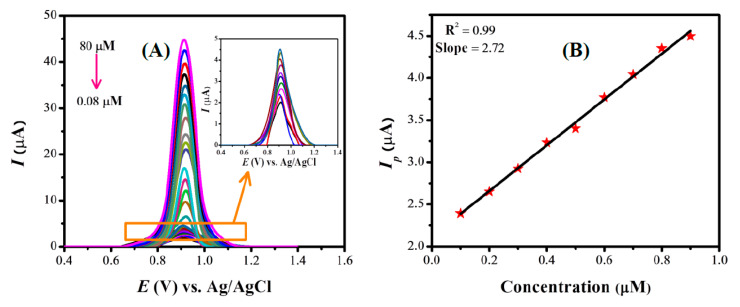
(**A**) SWVs for different concentrations of ST varying from 80–0.08 µM in PBS (pH 6); (**B**) linearity plot for 0.9 µM to 0.1 µM concentrations of ST.

SWV was used to evaluate the sensitivity of the fabricated COOH-*f*MWCNTs/Fe-ZnO/GCE sensor by varying the concentration from higher (80 µM) to lower concentrations (0.08 µM) under optimized experimental conditions, i.e., 0.1 M PBS (pH 6), −0.2 V E_d_, 10 s D_t_. The recorded SWVs are depicted in Figure 5A. As the current is proportional to the concentration, a reciprocal trend in the current response was observed with a variation in concentration. From the lower concentration data of ST, the linearity plot was established as shown in Figure 5B. The limit of quantification (*L_Q_*) of 7.8 nM and the limit of detection (*L_D_*) of 2.3 nM were estimated by employing Equations (4) and (5).

(4) $$L_{Q}=\frac{10\sigma }{m}$$

(5) $$L_{D}=\frac{3\sigma }{m}$$

Here, *σ* is the standard deviation, which was determined by the anodic peak current of the blank solution (sixteen runs), and *m* represents the value of slope determined from the linearity plot.

#### 3.6.2. Evaluation of the Stability of the Designed Sensing Scaffold

To scrutinize the accuracy and precision of the designed COOH-*f*MWCNTs/Fe-ZnO scaffold, the reproducibility and repeatability of the modified GCE were investigated. The stability of the presented sensor was assessed by measuring its electrochemical response in the presence of ST dye while being subjected to pre-optimized experimental parameters. 

The reproducibility of the developed sensor was inspected using SWV analysis by fabricating six different COOH-*f*MWCNTs/Fe-ZnO/GCEs. Figure S7A depicts the SWV responses of ST (30 µM) in 0.1 M PBS. The oxidation signals of all six electrodes were found to be nearly identical validating the superb reproducibility (%RSD = 0.28) of the presented sensor. To investigate the repeatability of the proposed sensor, COOH-*f*MWCNTs/Fe-ZnO/GCE was dipped in a PBS solution of pH 6 for different timespans, and then voltammetric measurements were executed. Very consistent peak signals were observed up to 36 h, as illustrated in Figure S7B. Conclusively, these findings revealed that COOH-*f*MWCNTs/Fe-ZnO/GCE demonstrated repeatable (%RSD = 0.72) and reproducible outcomes, indicating the superior quality of our proposed sensor.

Textile factories discharge numerous contaminants such as dyes, metals, and other toxic materials. The most prevalent metal ions identified in textile effluents include Cd (II), Cr (VI), Pb (II), and Zn (II), which are discharged in substantial quantities by the textile industry. These metals are used to manufacture the color pigments used in textile dyes. It is conceivable that these species could have an impact on the sensor’s sensing performance. To determine the impacts of interfering agents on the oxidation peak intensity of the analyte, realistic scenarios were simulated by separately adding 0.1 mM of different metal ions and dyes to the 30 µM ST solution. Figure S7C shows SWVs of 30 µM ST recorded in the presence of different interfering agents. These results show that the SWV signal of ST is not appreciably affected in the presence of many-times-higher concentrations of interfering agents (%RSD = 2.02). As can be seen from the plot, when dye solutions were tested as interfering agents, additional signals of dyes also appeared alongside the distinctive signal of ST, but they had no discernible impact on the signal of the analyte. These findings can be ascribed to the high affinity of the analyte toward the sensing scaffold in comparison to the interfering agents, as well as the poor solubility of the designed sensor in an aqueous medium.

### 3.7. Photodegradation of ST

After analyzing the electrocatalytic behavior of ZnO and Fe-ZnO for the detection of ST, the photocatalytic efficacy was also evaluated by performing photodegradation of ST under solar light illumination. For comparison to select the most potent photocatalyst between ZnO and Fe-ZnO, the photodegradation of ST was monitored using UV-visible spectroscopy. The photocatalytic efficacy was assessed by adding a 2 mg dose of each ZnO and Fe-ZnO in 40 mL of the ST solution (80 µM) under neutral pH. Photocatalytic degradation was monitored by recording the absorption spectra (Figure S8A,B), as well as by analyzing the color change. The experimental findings showed the enhanced photocatalytic efficacy of Fe-ZnO in comparison to ZnO, which degrade ≈ 92% of ST in the given period (Figure S8C). This increased performance of Fe-ZnO can be ascribed to the reduction of the band gap (3.25 eV → 3.11 eV) and less agglomeration, which allows the provision of more surface area.

#### 3.7.1. Influence of pH on Photocatalytic Degradation

The pH of the solution is an influential factor that significantly affects the efficiency of a photocatalyst. The pH of the solution profoundly impacts the surface characteristics of the photocatalyst such as the position of the band edge, the size of particles’ aggregation, and the charge of the catalyst. The impact of pH on the photodegradation of ST was examined by conducting experiments at pH ranging from 3 to 11. Before the experiments, the pH of the ST solution was maintained by using 0.1 M NaOH and 0.1 M HCl. The percentage elimination of ST was found to be 22% for pH 3, which increased to 99% for pH 11 (Figure S9A). The comparative kinetic plots of photodegradation of ST at different pH are presented in Figure S9B, whereas the extent of degradation and rate constants are summarized in Table S5. ST degrades most quickly in basic conditions. The basic environment favors the deprotonation of the catalyst surface, and as a result, the negatively charged surface efficiently uptakes the cationic dye ST, resulting in faster degradation. Similarly, in basic pH, more hydroxyl ions are available, which produce more hydroxyl radicals (∙OH) at the photocatalyst surface, which boost the photodegradation rate. At neutral and basic pH, the ∙OH radicals are supposed to be the main oxidizing species, whereas at acidic pH, holes are regarded as the predominant species. The enhanced photodegradation of ST at basic pH indicates that ∙OH radicals are the major oxidizing species involved in the degradation process. Thus, pH 11 was found to be the optimal value for photodegradation of ST, which removed ≈99% dye in 60 min.

#### 3.7.2. Electrochemical and Spectroscopic Monitoring of the Photocatalytic Degradation of ST

Under the optimized value of pH 11, the photodegradation of ST was monitored using UV-visible spectroscopy and electrochemical methods (SWV). The degradation experiment was performed by adding 2 mg of the Fe-ZnO dose to 40 mL of the ST solution. The solution was illuminated under direct solar light while being continuously stirred to ensure that the photocatalyst was evenly dispersed. A 2 mL solution was taken at different time intervals, and degradation was monitored using electrochemical and UV-visible spectroscopic techniques.

**Figure 6:**
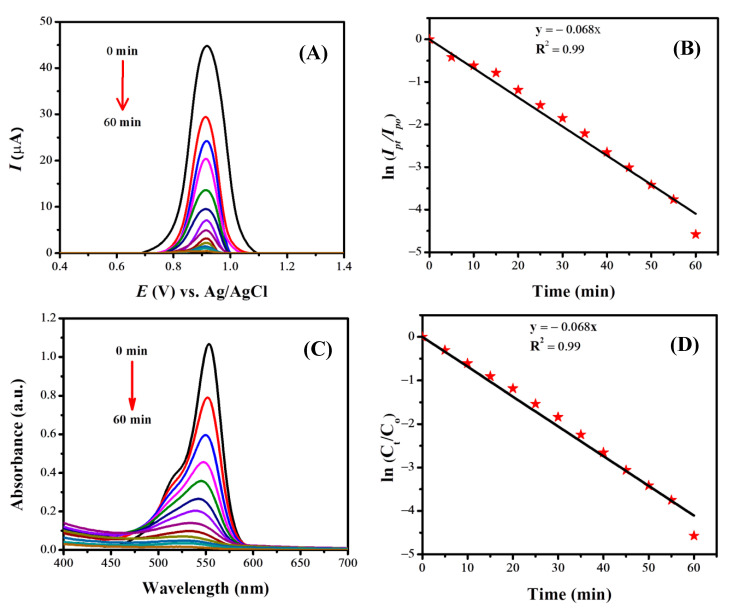
(**A**) SWVs of ST photodegradation recorded at various times using designed sensor; (**B**) kinetics plot of photodegradation of ST constructed from SWV data; (**C**) UV-visible monitoring of photocatalytic degradation of ST; (**D**) pseudo-first-order kinetics plot of ST photodegradation.

For the electrochemical monitoring of the photodegradation of ST, the developed COOH-*f*MWCNTs/Fe-ZnO/GCE sensor was used. To the modified GCE, a 10 µL droplet of dye obtained at regular time intervals from the solution was cast on it. Then, recording the SW voltammograms using the optimized conditions (PBS pH 6, E_d_—0.2 V, and D_t_ 10 s), the degradation of ST was investigated. Figure 6A depicts the SW voltammograms of ST recorded at different time intervals. The current signal of dye is proportional to its concentration, therefore as the degradation of dye proceeds, a decline in the current intensity was observed due to the diminishing of dye with time as evident from color fadedness. 

The percentage degradation of ST was determined using the following formula:

(6) $$\%Degradation=\frac{{Ip}_{0}-{Ip}_{t}}{{Ip}_{0}}\times 100$$

where *Ip_o_* corresponds to the maximum peak current observed at the start of the degradation process, and *Ip_t_* denotes the peak current observed at a different time interval. The variation of percentage degradation with the course of the reaction is shown in Figure S10A. The kinetic behavior of ST degradation was also investigated using the SWV data. The degradation data fit best in the pseudo-first-order kinetic equation as depicted in Figure 6B. The kinetic equation of the pseudo-first-order reaction in terms of current can be written as:

(7) $$ln\frac{{Ip}_{t}}{{Ip}_{0}}=-kt$$

The rate constant for the photodegradation of ST has a value of 0.068 min^−1^, determined from the value of the slope.

UV-visible spectroscopy was also employed to monitor the photodegradation of ST. Absorption spectra of ST degradation recorded at different time intervals are illustrated in Figure 6C. The absorption of ST decreases with the passage of time showing the diminishing concentration of ST in the solution. The absorbance spectra were also used to estimate the extent of degradation (%degradation) using the formula shown in Equation (8).

(8) $$\%Degradation=\frac{C_{0}-C_{t}}{C_{0}}\times 100$$

where *C_0_* represents the absorbance at a time equal to 0 min (corresponding to the time the experiment commenced), and *C_t_* symbolizes the absorbance recorded at time *t*. The graph in Figure S10B shows the %degradation of ST over time. Kinetic analysis of ST degradation was also carried out utilizing UV-visible spectroscopic data. The data fit best according to the pseudo-first-order kinetic equation as can be seen in Figure 6D. The kinetic parameter (*k*) of the reaction was calculated using Equation (9).

(9) $$ln\frac{C_{t}}{C_{0}}=-kt$$

**Figure 7:**
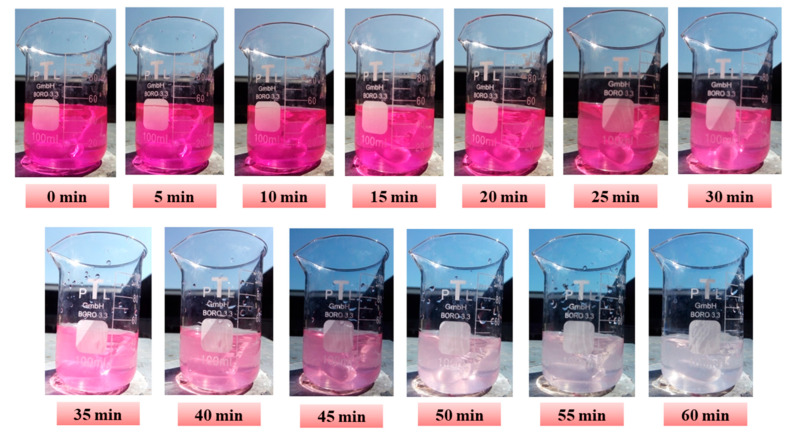
Visual evidence of photodegradation of ST using Fe-ZnO under solar-light illumination.

The slope value of the plot between *ln (C_t_/C_0_)* and time shows the rate constant of ST degradation, which is equal to 0.068 min^−1^. The percentage degradation and rate constant findings obtained from electrochemical and UV-visible techniques are comparable. Moreover, by monitoring the photodegradation of ST employing voltammetric and spectroscopic techniques, it was also monitored visually. The color variation of the dye with time was recorded by taking photographs using a mobile phone. Figure 7 displays the change in color of the dye, demonstrating the deterioration of the dye over time.

To analyze the photocatalytic efficiency of Fe-ZnO NPs, the degradation of ST was examined without a catalyst under direct sunlight illumination. The photodegradation of ST without a catalyst was performed for 1 h using an 80 µM concentration of dye in a medium of pH 11. Figure S11A depicts the UV-Vis spectra of ST recorded at different time intervals. No appreciable degradation was noticed for 1 h. ST was photodegraded only up to 3.6% without Fe-ZnO (Figure S11B). When Fe-ZnO NPs were used as the photocatalyst for the degradation of ST, up to 99% degradation was observed within the same time span of 1 h (Figure 6C), which confirms the excellent photocatalytic performance of Fe-ZnO for ST degradation.

#### 3.7.3. Stability of the Photocatalyst

**Figure 8:**
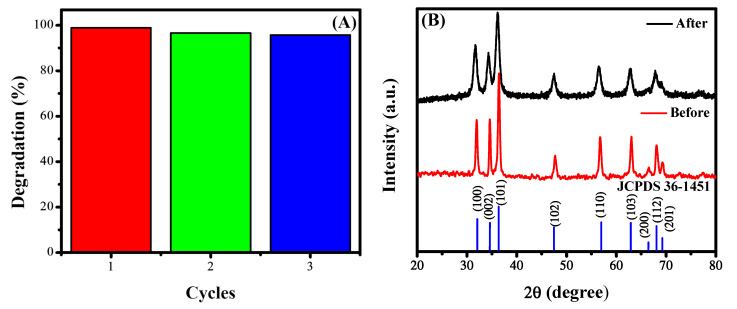
(**A**) Recycle catalytic performance of Fe-ZnO photocatalyst; (**B**) XRD pattern of Fe-ZnO recorded before and after the photocatalytic degradation of ST.

An ideal photocatalyst should maintain its catalytic ability even after multiple usages. To analyze the stability of Fe-ZnO NPs, a reusability experiment was performed using 40 mL of the 80 µM ST solution of pH 11. After the degradation experiment, the catalyst was recovered by centrifuging the dye solution at 6000 rpm for 10 min. The degradation experiment was recorded using UV-Vis spectroscopy. Fe-ZnO NPs demonstrated moderately stable photocatalytic performance in three consecutive photocatalytic degradation experiments. The loss of photocatalytic activity from approximately 99% (Figure S12A) to 95.7% (Figure S12C) occurred after three uses, which may be due to the occupation of the catalyst surface sites with dye molecules and their degraded products or may be due to the photo corrosion of Fe-ZnO NPs. Other factors such as material loss during the recovery process and the aggregation of NPs may result in a decrease in the photocatalytic performance. The stability of Fe-ZnO NPs was also investigated using XRD analysis. Figure 8 shows the XRD pattern of Fe-ZnO NPs recorded after the photocatalytic degradation experiment. The XRD results suggest that there is no significant change in the structure and crystalline phase after multiple usages. The crystallinity of Fe-ZnO becomes lower after its third usage. The significant decrease in the intensity of diffraction peaks corresponding to 200 and 201 planes in comparison to other peaks suggest the prominent role of these planes in the catalytic activity of the synthesized catalyst. 

#### 3.7.4. Proposed Mechanism of ST Photodegradation

**Figure 9:**
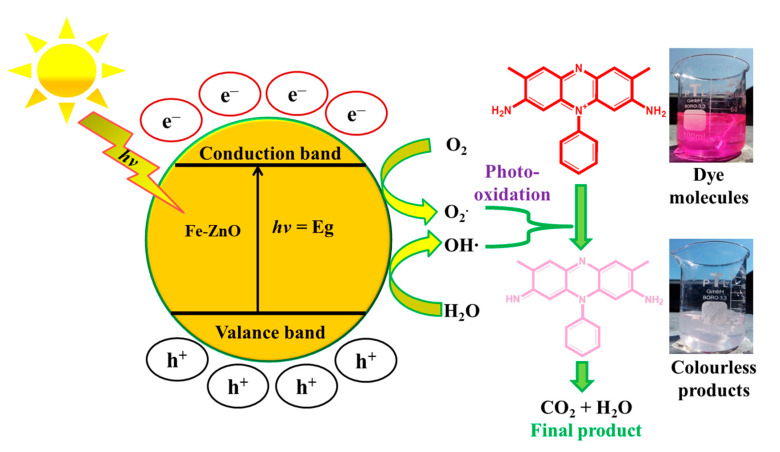
Proposed pathway for the solar light-assisted degradation of ST using Fe-ZnO.

A possible photodegradation mechanism of ST using Fe-ZnO is depicted in Figure 9. When the ST solution containing Fe-ZnO was exposed to solar light, the photons with energy greater or equal to the band gap energy of Fe-ZnO causes the excitation of electrons from the valance band to the conduction band, which results in the photogeneration of holes (h^+^) at their positions. After the photogeneration of electron–hole pairs, Fe-ZnO NPs serve as electron acceptors or donors for molecules in the surrounding medium. The interaction of holes with water causes the formation of the hydroxyl radical (OH∙), which is a strong oxidant (Equation (11)). Similarly, the superoxide anion radical ($O_{2}^{.-}$) is generated when the electron reduces the adsorbed oxygen (Equation (12)). The protonation of $O_{2}^{.-}$ leads to the formation of the hydroperoxyl radical (${HOO}^{.})\;$(Equation (13)), which results in hydrogen peroxide (H_2_O_2_) (Equation (14)), which ultimately dissociates to form OH∙. OH∙ is an extremely reactive oxidant that attacks the dye molecules present in the solution and adsorbed on the surface of the photoexcited catalyst. The breakdown of ST was assumed to occur through photo-oxidation by $O_{2}^{.-}$ and ${OH}^{.}$ radicals. The plausible mechanism of ST photodegradation is given below.

(10) $$Fe-ZnO+hv\;\to \;e^{-}+h^{+}$$

(11) $$H_{2}O+h^{+}\to \;H^{+}+{OH}^{.}$$

(12) $$O_{2}+e^{-}\;\to \;O_{2}^{.-}$$

(13) $$O_{2}^{.-}+H^{+}\;\to \;{HOO}^{.}$$

(14) $${HOO}^{.}+{HOO}^{.}\;\to \;H_{2}O_{2}+O_{2}$$

(15) $$H_{2}O_{2}\;\to \;2{OH}^{.}$$

(16) $${OH}^{.}+ST\;\to Photodegradation\;products$$

Table 1 reveals enhanced photocatalytic performance of Fe-ZnO NPs in comparison to reported Zn-based photocatalysts.

## 4. Conclusions

In summary, ZnO and Fe-ZnO NPs were prepared by the sol–gel method to explore their potential as photocatalysts and electrocatalysts for the photocatalytic degradation and voltammetric detection studies of ST dye. The synthesized materials were characterized by spectroscopic and voltammetric techniques. XRD analysis revealed the crystalline nature of the synthesized ZnO and Fe-ZnO NPs, whereas Tauc plots revealed a reduction in the band gap of ZnO after doping. A sensitive, selective, and stable electrochemical sensing scaffold comprising of COOH-*f*MWCNTs and Fe-ZnO modified GCE was prepared for the electrochemical detection and photocatalytic degradation monitoring of ST. Condition-optimization experiments were performed and the best electrochemical performance of COOH-*f*MWCNTs/Fe-ZnO/GCE towards the detection of ST was achieved with a PBS supporting electrolyte in a medium of pH 6, a deposition potential of −0.2 V, and a deposition time of 10 s. The nanomolar level limit of detection (2.3 nM), promising reproducibility (%RSD = 0.28), repeatability (%RSD = 0.72), and selectivity (%RSD = 2.02) of the designed sensor are the prominent attributes of ST detection. While ZnO showed good photocatalytic performance, Fe-ZnO NPs demonstrated even better performance toward the photocatalytic degradation of ST. The extent of degradation increased by increasing the pH of the solution with 99% degradation at pH 11 with a rate constant of 0.068 min^−1^. The diminishing color over time in the presence of a catalyst provided visual evidence of the ST photocatalytic degradation. According to the findings of both electrochemical and UV-visible spectrophotometric investigations, the breakdown of ST follows pseudo-first-order kinetics. The present study reports voltammetric detection and degradation monitoring by employing COOH-*f*MWCNTs/Fe-ZnO/GCE. However, in the future, more semiconducting NPs with different morphologies such as nanoflowers, nanobelts, nanofibers, nanotubes, etc., can be utilized in synergism with COOH-*f*MWCNTs for ultrasensitive picomolar and even femtomolar-level detection of ST and other dyes.

## Figures and Tables

**Table 1:** Comparison of performance of Fe-ZnO for photodegradation of ST with those reported in the literature.

S. No.	Photocatalyst	Catalyst Dose (mg)	% Removal	Irradiation Time (min)	Light Source	References
1	Maize biochar-ZnO nanocomposite	5	87	60	500 W Xe lamp	[56]
2	Zn0	100	85.29	90	UV light	[55]
3	Ag decorated ZnO/rGO	-	100	80	Philips white LED	[57]
4	ZnS	-	51	40	Xe lamp	[52]
5	Zn_0.98_Mn_0.02_S, Zn_0.94_Ni_0.06_S and Zn_0.90_Cu_0.10_S	-	85.4–93.2	-	100 W mercury lamp	[58]
6	Ag-doped ZnO	50	74.11	20	Sunlight	[59]
7	Fe-ZnO	2	≈99	60	Sunlight	This work

## Data Availability

Data are available in this manuscript and its supporting information.

## Supplementary Materials

The following supporting information can be downloaded at: https://www.mdpi.com/article/10.3390/nano13152218/s1. Table S1: The chemicals/reagents and their specifications. Scheme S1: Experimental setup for voltammetric detection of ST. Table S2: Elemental composition of ZnO and Fe-ZnO NPs estimated from EDX analysis. Figure S1: Equivalent circuit used for EIS parameter calculation. Table S3: Parameters obtained from EIS measurements. Table S4: Calculated surface areas and peak separations of working electrodes from the CV data of the redox probe K_3_[Fe(CN)_6_]. Figure S2: (A) Effect of various scan rates on the anodic peak current of ST in supporting electrolyte of PBS of pH 6.0; (B) Plot between *I_p_* vs. υ; (C) Peak current vs. square root of scan rate; (D) log *I_p_* vs. log υ. Figure S3: The impact of electrolyte media on the SWV peak current of 30 µM ST using COOH-fMWCNTs/Fe-ZnO/GCE. Figure S4: Influence of variation of pH of PBS (0.1 M) solution on the anodic peak current of ST. Figure S5: (A) Influence of deposition potential on ST oxidation peak current in PBS (pH 6) using COOH-fMWCNTs/Fe-ZnO/GCE at 5 s deposition time; (B) Plot of E_d_ (V) vs. *I_p_* (µA). Figure S6: (A) Impact of deposition time on the peak intensity of ST using COOH-fMWCNTs/Fe-ZnO/GCE; (B) Plot of *I_p_* (µA) vs. D_t_ (s). Figure S7: (A) Square wave voltammograms of ST showing the reproducibility of a fabricated sensor in PBS electrolyte; (B) SW voltammograms of ST showing the repeatability of the developed sensor in PBS (pH 6.0); (C) SWVs of 30 µM ST in the co-existence of various interfering agents. Figure S8: Photodegradation of ST using (A) ZnO; (B) Fe-ZnO under neutral pH condition; Graphical representation of (C) extent of photodegradation and (D) Pseudo-first order kinetics of photocatalytic degradation. Figure S9: (A) Graphical representation of the effect of pH on the extent of photocatalytic degradation of ST; (B) Kinetics of photodegradation of ST solution of different pH. Table S5: The value of extent of degradation and rate constants of photodegradation of ST using Fe-ZnO at different pH conditions. Figure S10: Plot of %age photodegradation of 80 µM ST using Fe-ZnO (A) from voltammetric data and (B) UV-visible data. Figure S11: (A) UV-Vis plot of photodegradation of ST without catalyst under direct sunlight illumination (B) Plot %age photodegradation of 80 µM ST without catalyst. Figure S12: UV-Vis spectra of photodegradation of 80 µM ST using Fe-ZnO (A) for first time (B) after recovery of catalyst for first time (C) after recovery of catalyst for second time.
