# Supplementary material for: Photoelectrocatalytic Detection and Degradation Studies of a Hazardous Textile Dye Safranin T
Source: Nanomaterials (Basel). 2023 Jul 30;13(15):2218. doi: 10.3390/nano13152218 (PMC10420668; doi:10.3390/nano13152218)
Supplement: Supplementary file 1 [file nanomaterials-13-02218-s001.zip › nanomaterials-2495311-supplementary.pdf]

Photoelectrocatalytic Detection and Degradation Studies of a Hazardous Textile Dye Safranin T

Table S1: The chemicals/reagents and their specifications.

S. No.	Chemicals/Reagents	Percentage purity (mass%)	Supplier
1.	Acetone	99	Riedel-deHaen
2.	Alizarin red S	99	Fluka
3.	Boric acid	>99	Sigma-Aldrich
4.	Cadmium chloride	99	Sigma-Aldrich
5.	Chromium nitrate	99	Sigma-Aldrich
6.	Dimethylformamide	99	Riedel-deHaen
7.	Disodium hydrogen phosphate dihydrate	>99	Merck
8.	Hydrochloric acid	37	Riedel-deHaen
9.	Methylene blue	99	Sigma-Aldrich
10.	Phosphoric acid	85	DaeJung
11.	Potassium chloride	99	Sigma-Aldrich
12.	Potassium hexacyanoferrate	>99	Sigma-Aldrich
13.	Potassium hydroxide	Extra pure	Sigma-Aldrich
14.	Potassium nitrate	99	DaeJung
15.	Sodium chloride	99	Merck
16.	Sodium dihydrogen phosphate monohydrate	>99	Merck
17.	Sulphuric acid	98	BDH

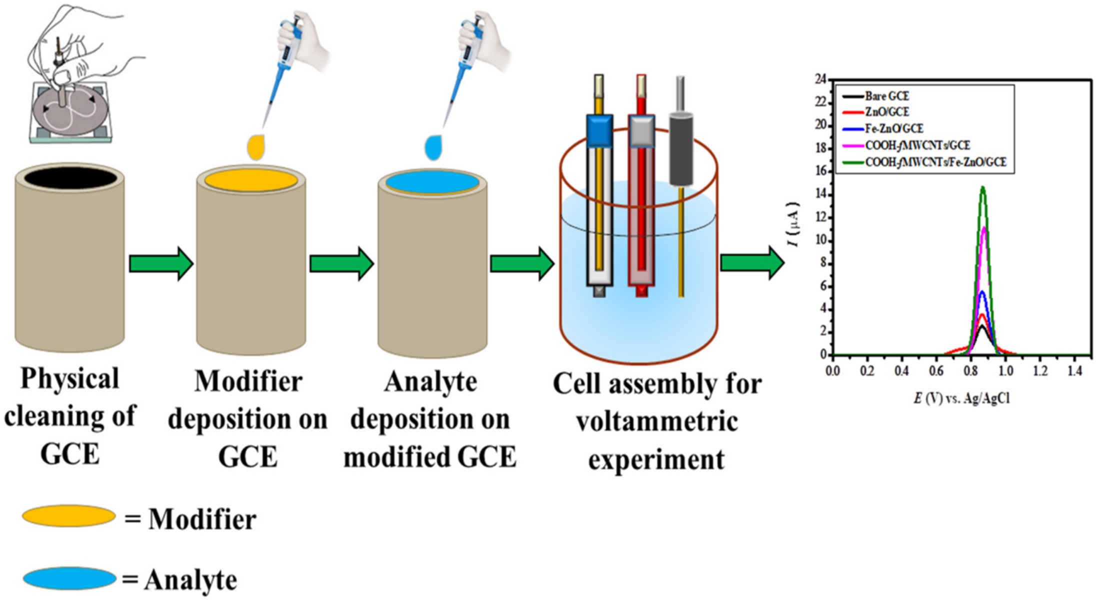

Scheme S1: Experimental setup for voltammetric detection of ST.

Table S2: Elemental composition of ZnO and Fe-ZnO NPs estimated from EDX analysis.

**Material**	**Chemical element (wt%)**			
	**Zn**	**O**	**Fe**	**C**
**ZnO**	70.24	19.36	--	10.14
**Fe-ZnO**	75	15.34	3.21	6.45

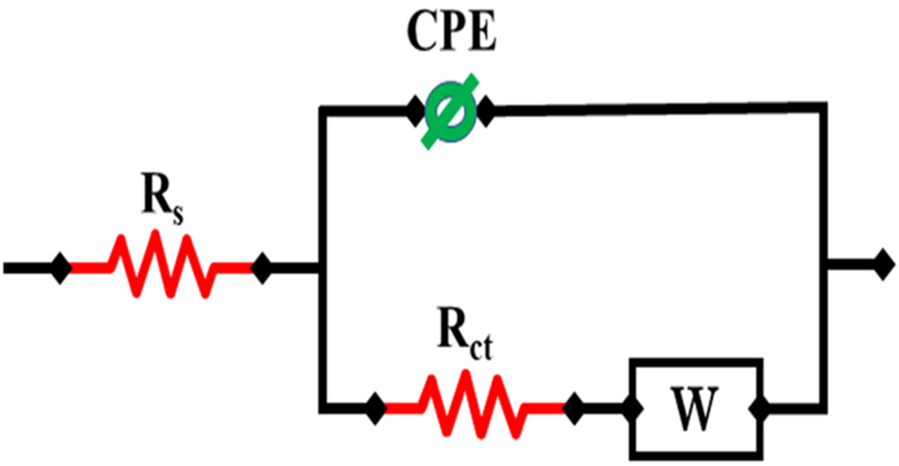

Figure S1: Equivalent circuit used for EIS parameter calculation.

Table S3: Parameters obtained from EIS measurements.

**Working electrode**	**R_s (Ω)**	**R_{ct} (Ω)**	**CPE (μF)**
Bare GCE	101	8030	1.96
ZnO/GCE	108	6650	2.19
Fe-ZnO/GCE	113	5480	3.20
COOH-*f*MWCNTs/GCE	110	2059	7.79
COOH-*f*MWCNTs/Fe-ZnO/GCE	107	395.3	1.09

Table S4: Calculated surface areas and peak separations of working electrodes from the CV data of the redox probe $K_3[Fe(CN)_6]$.

**Working electrode**	**Surface area (cm^2)**	**Peak separation (Δ*Ep*) (mV)**
Bare GCE	0.023	117
ZnO/GCE	0.028	107
Fe-ZnO/GCE	0.040	104
COOH-*f*MWCNTs/GCE	0.059	92
COOH-*f*MWCNTs/Fe-ZnO/GCE	0.092	65

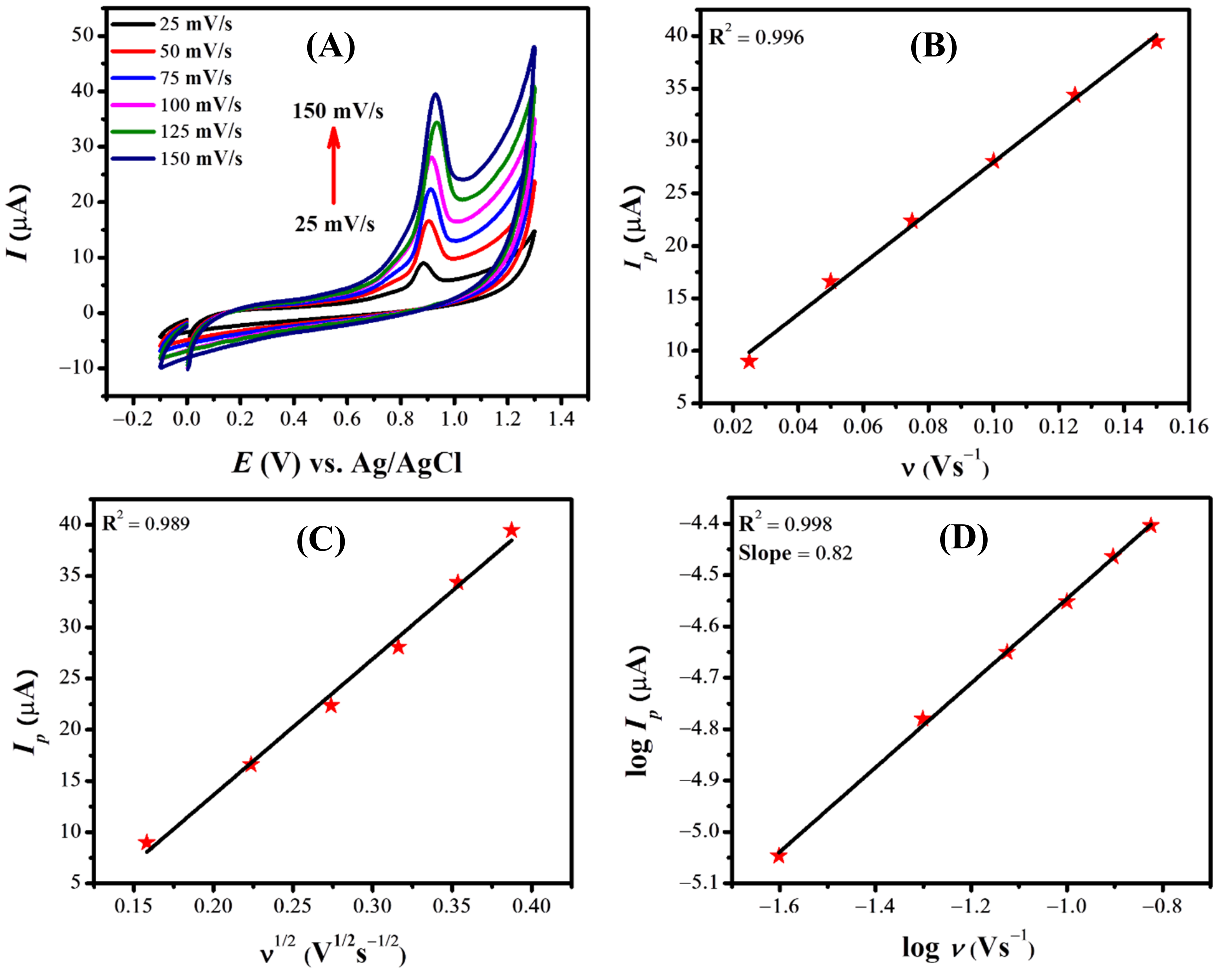

Figure S2: (**A**) Effect of various scan rates on the anodic peak current of ST in supporting electrolyte of PBS of pH 6.0; (**B**) Plot between I_p vs. υ; (**C**) Peak current vs. square root of scan rate; (**D**) log I_p vs. log υ.

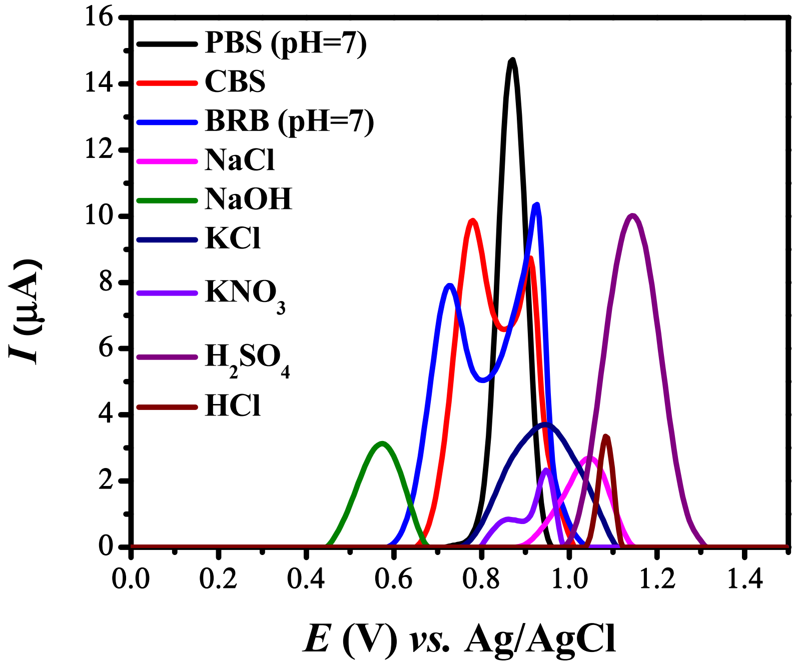

Figure S3: The impact of electrolyte media on the SWV peak current of 30 µM ST using COOH-*f*MWCNTs/Fe-ZnO/GCE.

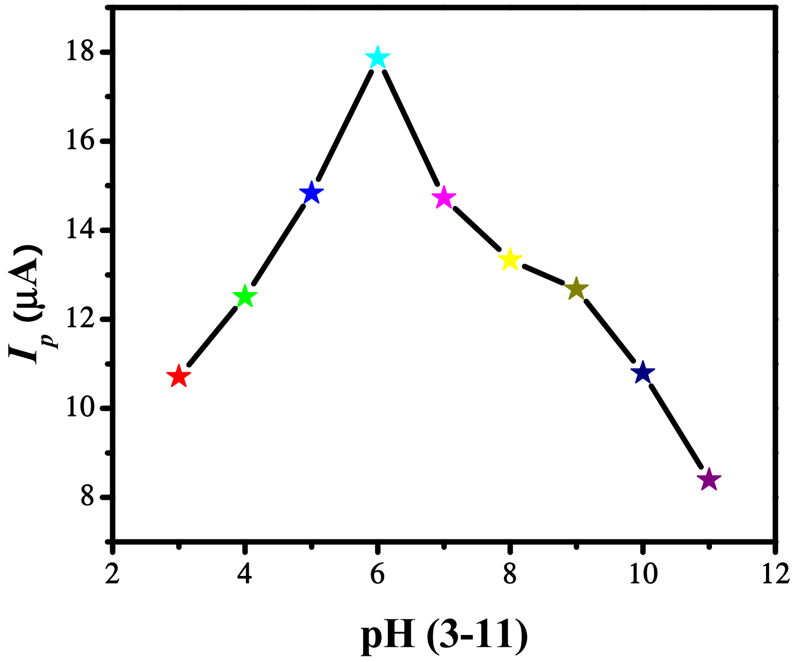

Figure S4: Influence of variation of pH of PBS (0.1 M) solution on the anodic peak current of ST.

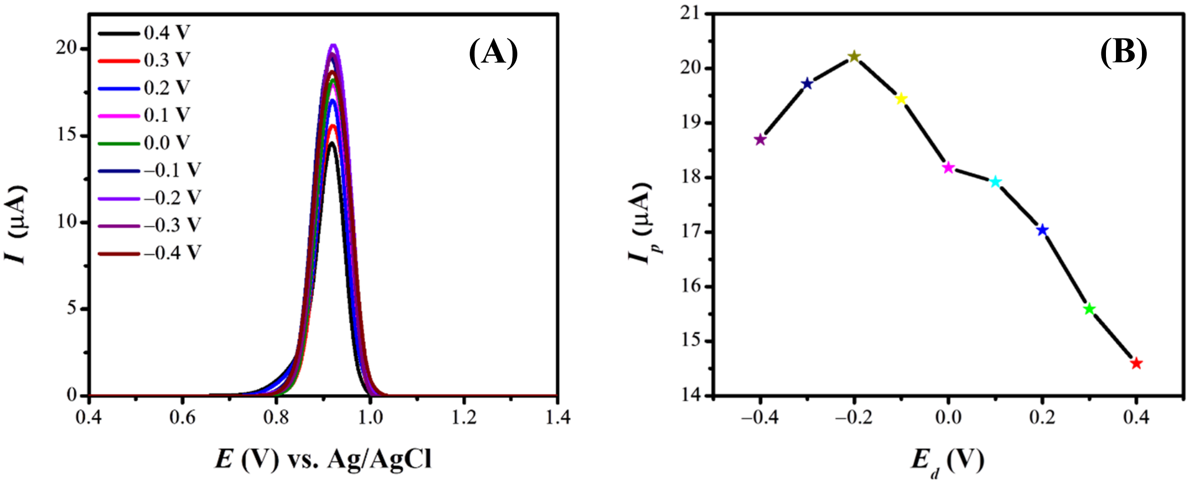

Figure S5: (**A**) Influence of deposition potential on ST oxidation peak current in PBS (pH 6) using COOH-*f*MWCNTs/Fe-ZnO/GCE at 5 s deposition time; (**B**) Plot of E_d (V) vs. I_p (μA).

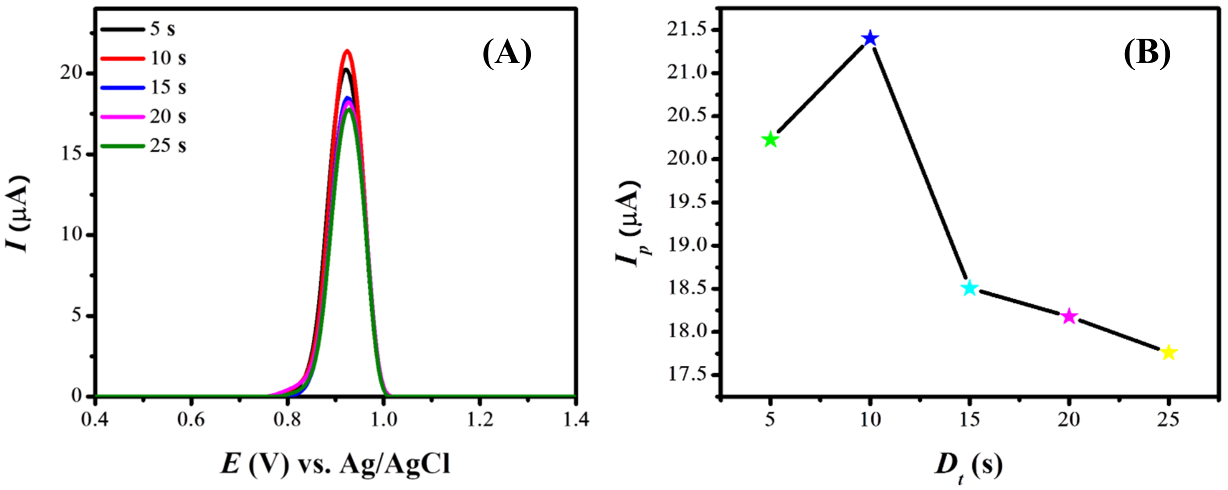

Figure S6: (**A**) Impact of deposition time on the peak intensity of ST using COOH-*f*MWCNTs/Fe-ZnO/GCE; (**B**) Plot of I_p (μA) vs. D_t (s).

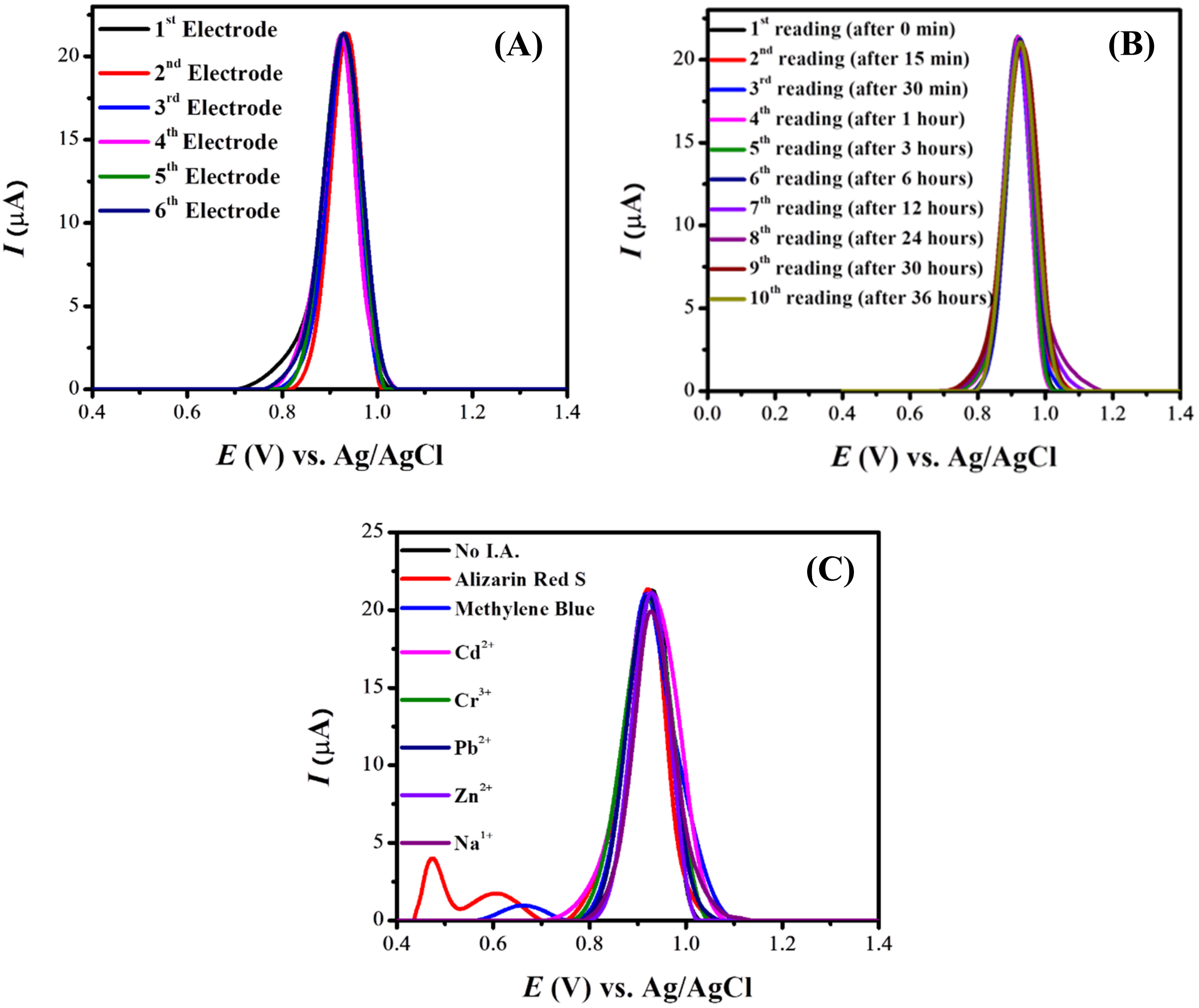

Figure S7: (**A**) Square wave voltammograms of ST showing the reproducibility of a fabricated sensor in PBS electrolyte; (**B**) SW voltammograms of ST showing the repeatability of the developed sensor in PBS (pH 6.0); (**C**) SWVs of 30 μM ST in the co-existence of various interfering agents.

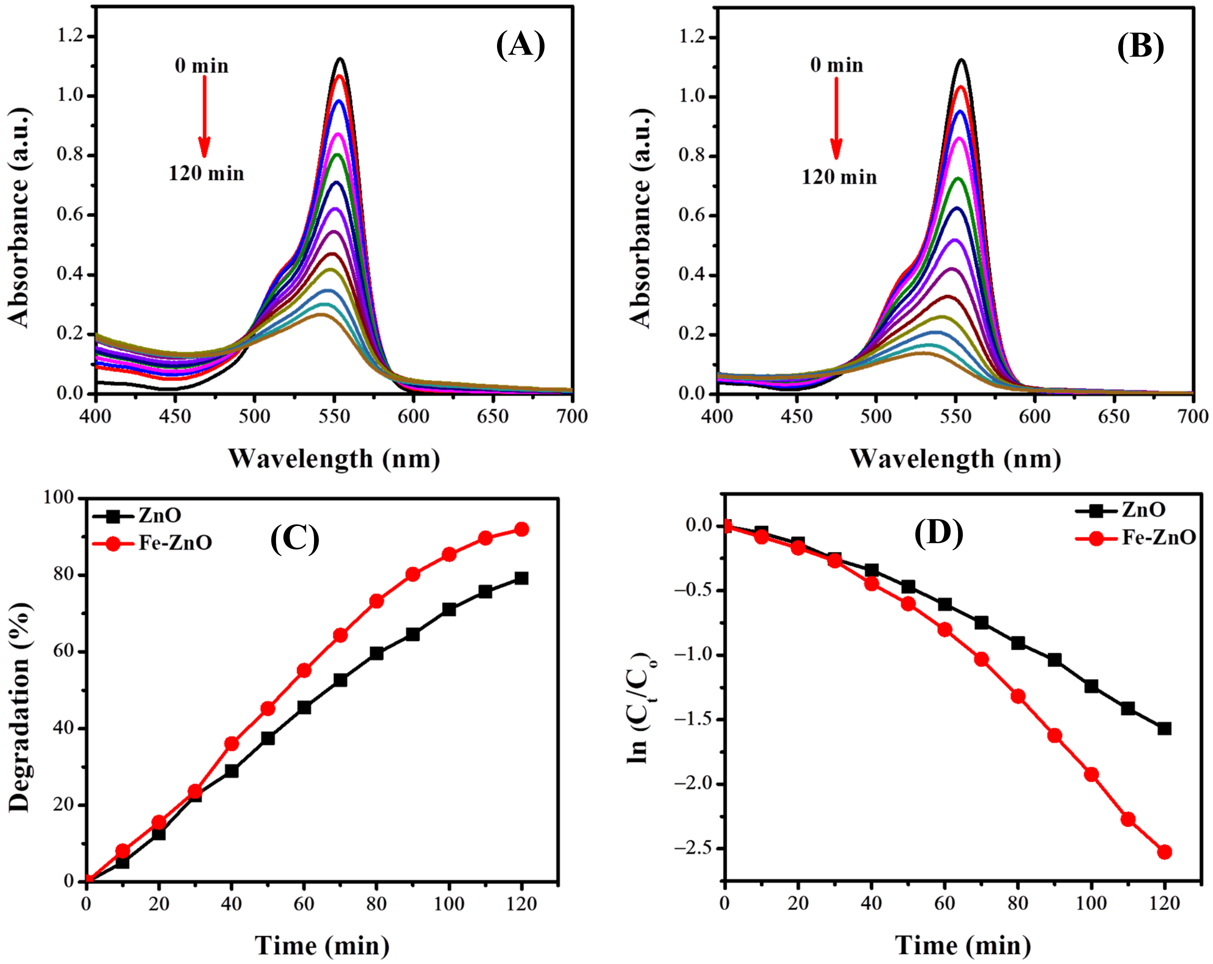

Figure S8: Photodegradation of ST using (**A**) ZnO; (**B**) Fe-ZnO under neutral pH condition; Graphical representation of (**C**) extent of photodegradation and (**D**) Pseudo-first order kinetics of photocatalytic degradation.

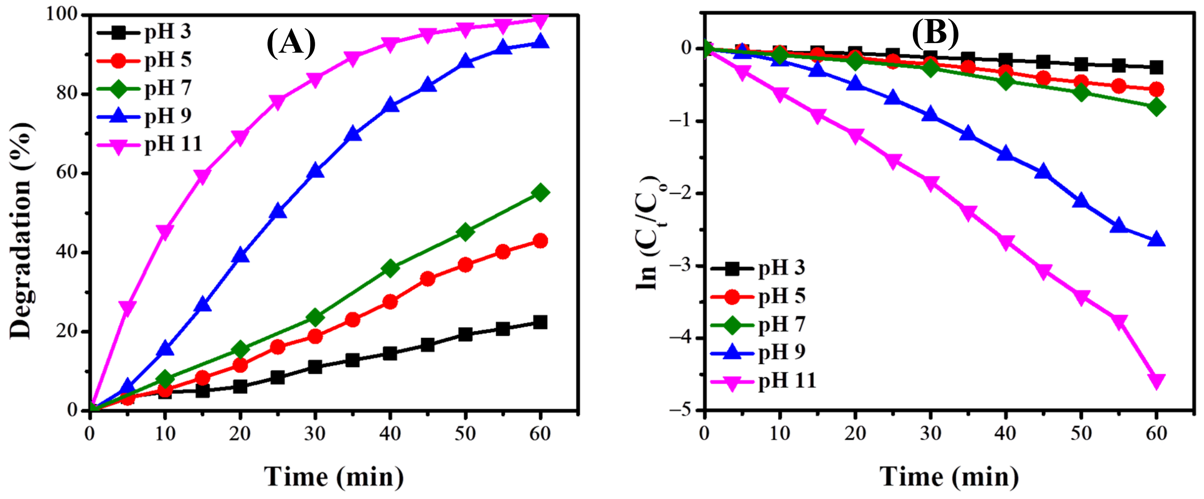

Figure S9: (**A**) Graphical representation of the effect of pH on the extent of photocatalytic degradation of ST; (**B**) Kinetics of photodegradation of ST solution at different pH.

Table S5: The value of the extent of degradation and rate constants of photodegradation of ST using Fe-ZnO at different pH conditions.

pH	Extent of degradation	Rate constant k = 10^{-2} (min^{-1})
3	22.37	0.41
5	42.97	0.87
7	55.16	1.19
9	92.97	3.93
11	98.97	6.84

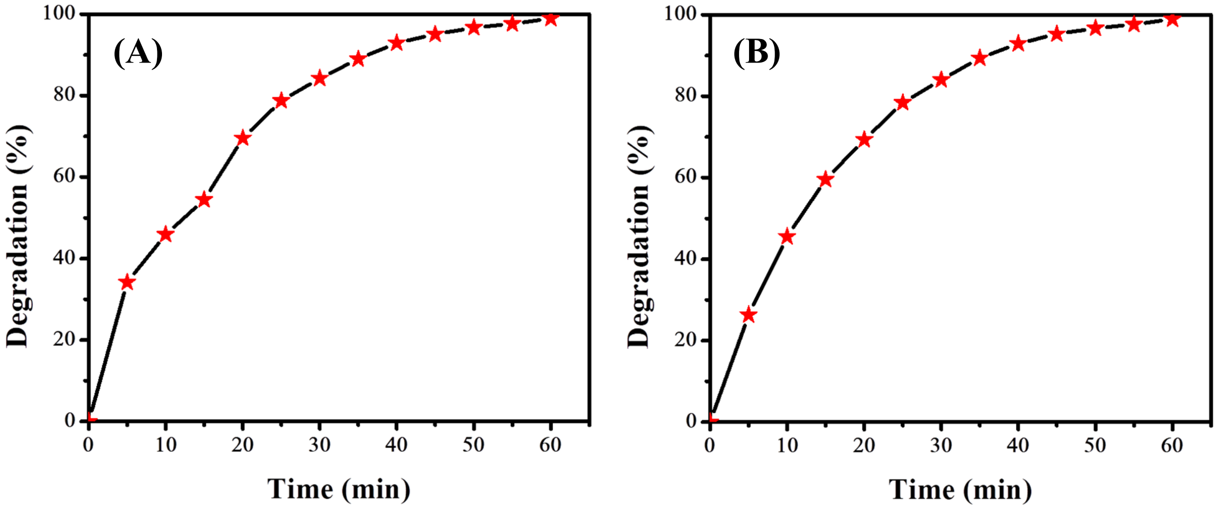

Figure S10: Plot of %age photodegradation of 80 μM ST using Fe-ZnO (**A**) from voltammetric data and (**B**) UV-visible data.

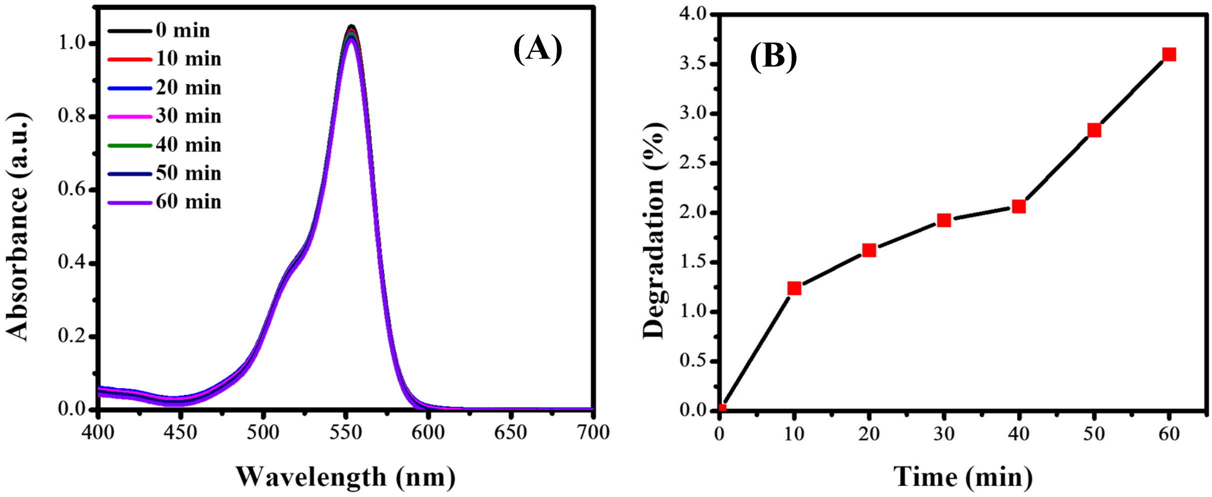

Figure S11: (A) UV-Vis plot of photodegradation of ST without catalyst under direct sunlight illumination **(B)** Plot %age photodegradation of 80 μM ST without catalyst**.**

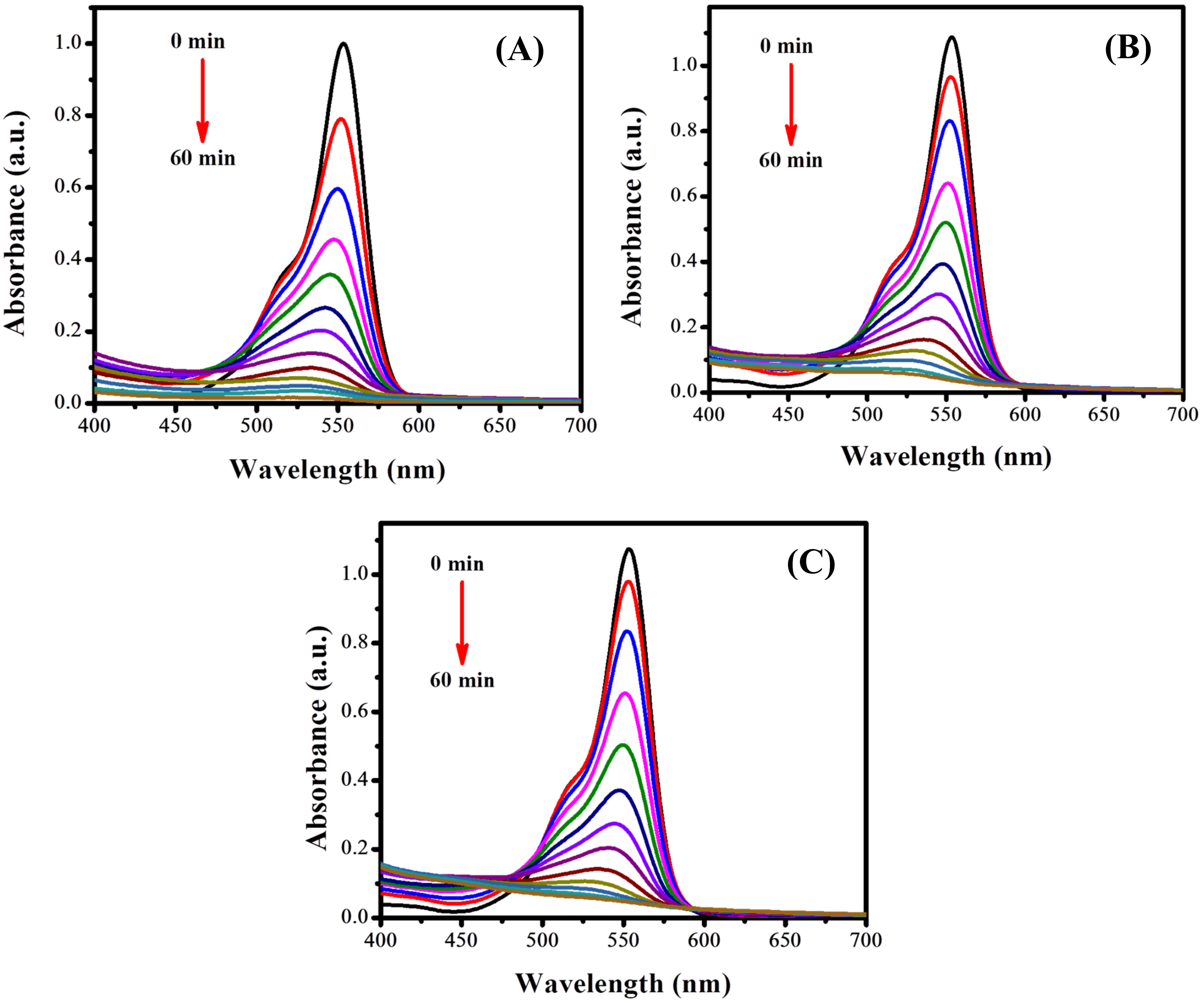

Figure S12: UV-Vis spectra of photodegradation of 80 μM ST using Fe-ZnO **(A)** for first time **(B)** after recovery of catalyst for fist time **(C)** after recovery of catalyst for second time.
